# Breeding without Breeding: Is a Complete Pedigree Necessary for Efficient Breeding?

**DOI:** 10.1371/journal.pone.0025737

**Published:** 2011-10-03

**Authors:** Yousry A. El-Kassaby, Eduardo P. Cappa, Cherdsak Liewlaksaneeyanawin, Jaroslav Klápště, Milan Lstibůrek

**Affiliations:** 1 Department of Forest Sciences, Faculty of Forestry, University of British Columbia, Vancouver, British Columbia, Canada; 2 Instituto Nacional de Tecnología Agropecuaria (INTA), Instituto de Recursos Biológicos, Hurlingham, Buenos Aires, Argentina; 3 Department of Dendrology and Forest Tree Breeding, Faculty of Forestry and Wood Sciences, Czech University of Life Sciences Prague, Praha, Czech Republic; University of Umeå, Sweden

## Abstract

Complete pedigree information is a prerequisite for modern breeding and the ranking of parents and offspring for selection and deployment decisions. DNA fingerprinting and pedigree reconstruction can substitute for artificial matings, by allowing parentage delineation of naturally produced offspring. Here, we report on the efficacy of a breeding concept called “Breeding without Breeding” (BwB) that circumvents artificial matings, focusing instead on a subset of randomly sampled, maternally known but paternally unknown offspring to delineate their paternal parentage. We then generate the information needed to rank those offspring and their paternal parents, using a combination of complete (full-sib: FS) and incomplete (half-sib: HS) analyses of the constructed pedigrees. Using a random sample of wind-pollinated offspring from 15 females (seed donors), growing in a 41-parent western larch population, BwB is evaluated and compared to two commonly used testing methods that rely on either incomplete (maternal half-sib, open-pollinated: OP) or complete (FS) pedigree designs. BwB produced results superior to those from the incomplete design and virtually identical to those from the complete pedigree methods. The combined use of complete and incomplete pedigree information permitted evaluating all parents, both maternal and paternal, as well as all offspring, a result that could not have been accomplished with either the OP or FS methods alone. We also discuss the optimum experimental setting, in terms of the proportion of fingerprinted offspring, the size of the assembled maternal and paternal half-sib families, the role of external gene flow, and selfing, as well as the number of parents that could be realistically tested with BwB.

## Introduction

Plant breeding, including tree improvement, typically follows the classical recurrent selection scheme, which is characterized by systematic and repetitive cycles of breeding, testing, and selection [1], [2]. These programs deal with multiple populations (e.g., base, breeding, and deployment) and large numbers of parents and offspring, planted over multiple sites and years, and requiring extensive monitoring and maintenance. Selection of elite genotypes for either further breeding and/or inclusion in production populations is commonly performed based on their breeding values, determined from the intra-class correlation among relatives produced from elaborate mating designs [3]. As breeding programs advance, the number of parents' increases and their genealogy overlaps, and mating designs become more elaborate and the time required for their completion become real breeding programs' limiting factors [4]. To alleviate the efforts associated with generating offspring with complete pedigree information, specifically for early generation testing, forest geneticists have adopted simplified protocols, ranging from those not requiring a pedigree (e.g., bulk samples from natural populations known as provenance testing [5] to those with incomplete pedigrees (e.g., open-pollinated [6] or polycross mating [7]). Data analyses with incomplete pedigrees often require invoking and/or accepting un-testable assumptions related to the genetic constitution of the tested families and the numbers of male parents involved in their formation, as well as their proportionate contributions. Since these assumptions are not inordinately realistic in practice, the resulting genetic parameters and their associated inferences are often biased, ultimately leading to various degrees of inaccuracy and inefficiency [8]–[10].

The availability of affordable, highly informative DNA markers, coupled with the development of sophisticated pedigree reconstruction methods, has enhanced their utility in converting incomplete pedigree trials into (effectively) complete trials, thus eliminating the pitfalls associated with the invocation of unfulfilled assumptions [11]. Lambeth *et al.* [11] initiative of converting the polycross mating design's incomplete pedigree to complete made proper quantitative genetic analyses possible and the method was repeatedly evaluated for several species [12]–[16]. El-Kassaby *et al.* [13] and El-Kassaby and Lstibůrek [17] capitalized on the restricted maximum likelihood-based “animal model” [18] capability of analysing unbalanced and incomplete pedigree data, along with pedigree reconstruction (tantamount to paternity assignment), to introduce the concept of “Breeding without Breeding (BwB).” The basic idea of BwB is to combine the use of offspring with incomplete pedigree information (an entire open-pollinated test) with a subset of offspring with complete pedigree information, to construct both parental and offspring breeding values, thus incorporating backwards, forwards, and combined selection into an efficient breeding framework [13], [17]. Most of the DNA fingerprinting effort is dedicated to a subset of the offspring from a small number of known maternal parents (seed donors) to generate information about the entire population (maternal and paternal parents, as well as offspring) after reassembling paternal half-sib families from the pedigree reconstruction of the fingerprinted subset. Pedigree reconstruction permits connecting the entire parental population (sampled or not) through their shared offspring thus allowing the implementation of classical quantitative genetics analyses [18].

Here we experimentally demonstrate the utility, the increased precision of genetic parameters estimation, and increased accuracy of predicted breeding values, hence the effectiveness of the “Breeding without Breeding” concept, using open-pollinated offspring from 15 of 41 parents in a western larch (*Larix occidentalis* Nutt.) “breeding population.” We compared the performance of the combined incomplete (half-sib: HS) + complete (full-sib: FS) analysis to that of both the incomplete and complete pedigree designs. Finally, we illustrate the optimum experimental efforts needed for the successful implementation of BwB and discuss the role of factors such as external gene flow, expansion of the test population (i.e., the number of tested parents), and the size of half- or full-sib family needed for accurate genetic parameter determination.

## Results

### Pedigree Reconstruction/Mating Design Assembly

**Figure 1:**
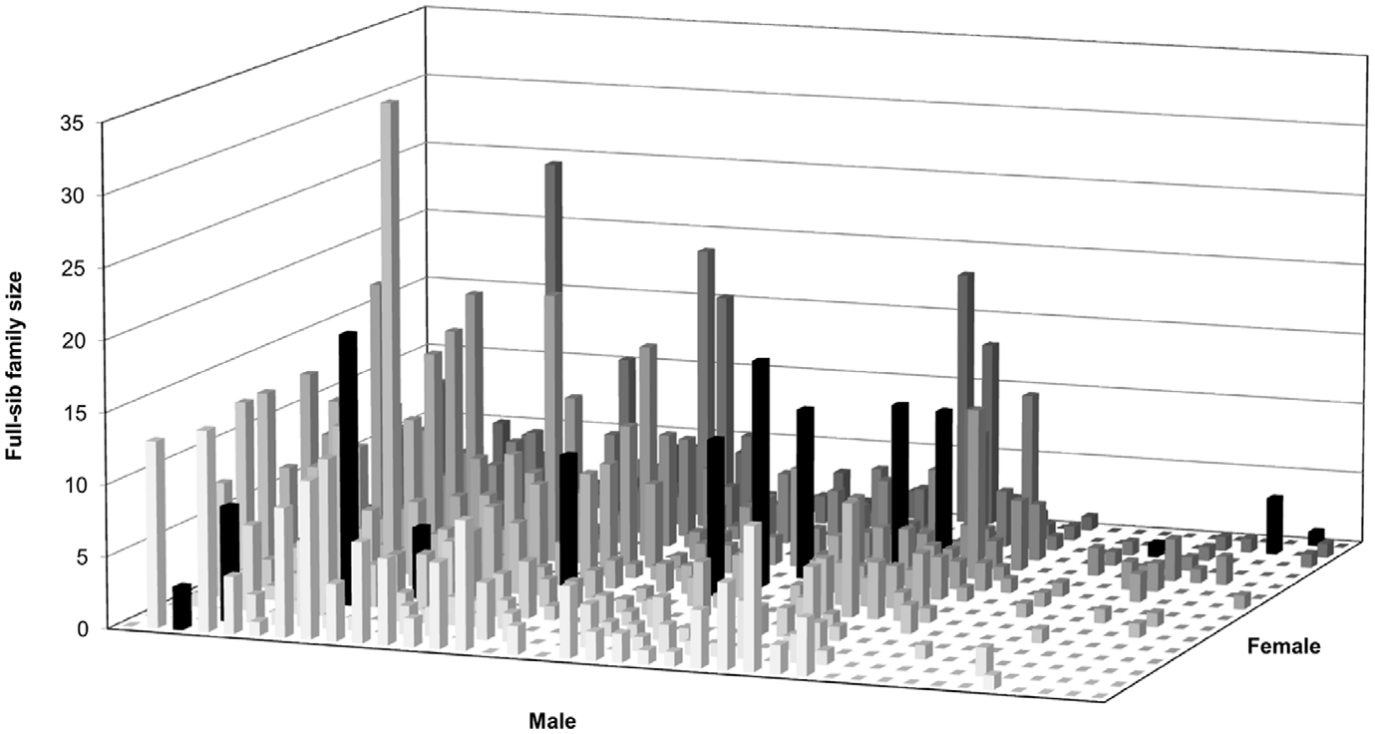
Pedigree reconstruction results showing the formation of full-sib families nested within the maternal and paternal half-sib families (black bars represent selfing).

The partial pedigree reconstruction allowed direct estimation of gene flow, selfing rate, male reproductive success, and the number and/or size of maternal and paternal half-sib families on the individual as well as the population level (Figure 1). With 95% confidence, 1,419 out of 1,538 (92.3%) fingerprinted offspring were assigned to male parents within the orchard (Figure 1). The remaining 119 paternally unassigned offspring were identified as the product of introgression from an adjacent orchard, suggesting a pollen immigration rate of 7.7%. In addition, a total of 113 individual offspring resulted from selfing (average: 7.4%), ranging from 0.0 to 26.8% among seed donors, reflecting the 15 maternal parents propensity variation to selfing. This variability could be caused by maternal parents' pollen shed and receptivity period synchrony differences.

Pedigree reconstruction resulted in the formation of 349 full-sib families, nested within the 15 maternal and 38 paternal half-sib families, respectively, indicating that three out of the orchard's potential 41 male parents did not participate in pollination, at least of these 15 maternal parents, most likely due to their recent introduction to the seed orchard population (pers. observation). The 15 maternal half-sib families had an average size of 283.9 (range: 222–397) and the 38 paternal half-sib families had an average size of 37.3 (range: 1–193 among the 38 recovered paternal sibships), the latter evidently reflecting male fecundity variation within the orchard. There was an apparently high correlation between the difficult to assess male reproductive investment (male strobili production) and male reproductive success (determined by paternity analysis [19] (*r* = 0.87; *P*<0.001).

The reconstructed pedigree formed a structured mating design, which we used to generate quantitative genetic parameters for the complete pedigree model (FS), and was used in concert with the non-fingerprinted individuals within each of the 15 HS families to form a combined pedigree model, consisting of half- and full-sib families (HS+FS) (see below). A minimum paternal half-sib family size threshold of six individuals was established for inclusion in quantitative genetic analyses. Seven male parents did not meet this threshold, but two were retained, because they were also represented as seed-donors, thus far exceeding the established minimum family size threshold.

### Estimation of Quantitative Genetic Parameters

**Figure 2:**
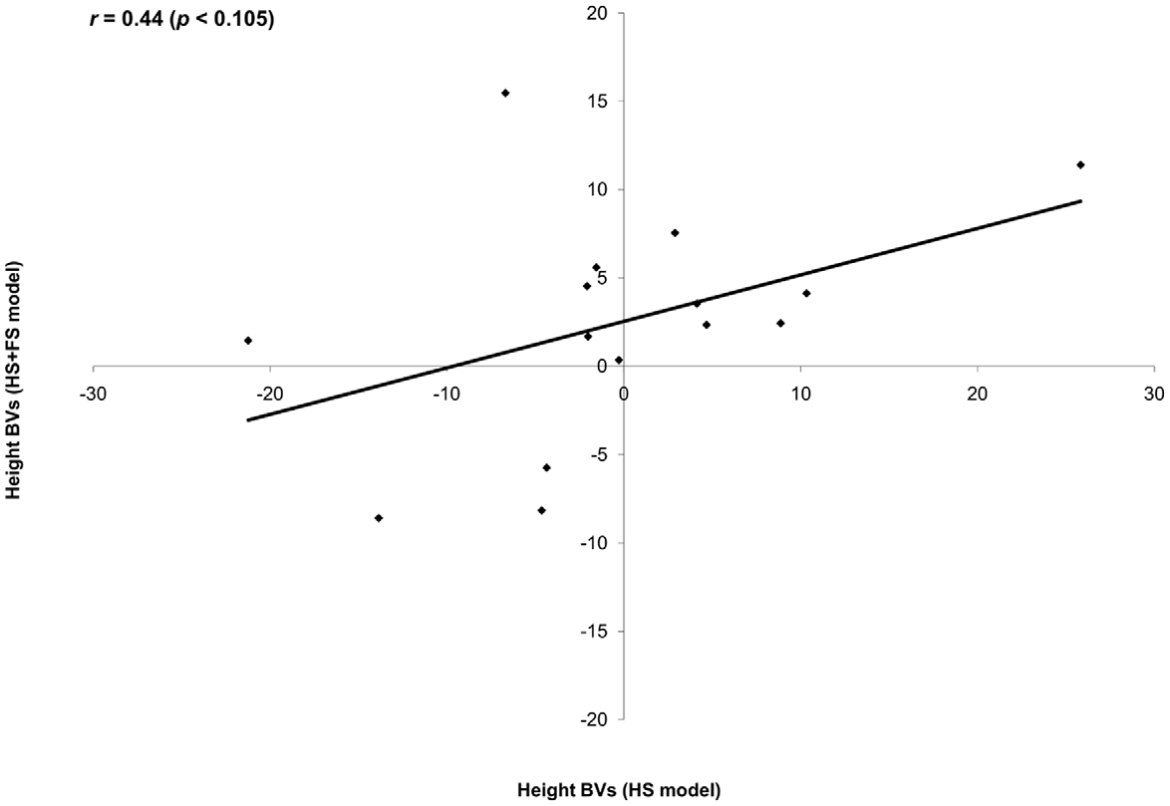
Scatter plot of predicted breeding values for parents from the two incomplete pedigree models (HS and combined FS+HS). Pearson correlation (*r*) is in the left corner of the graph.

**Figure 3:**
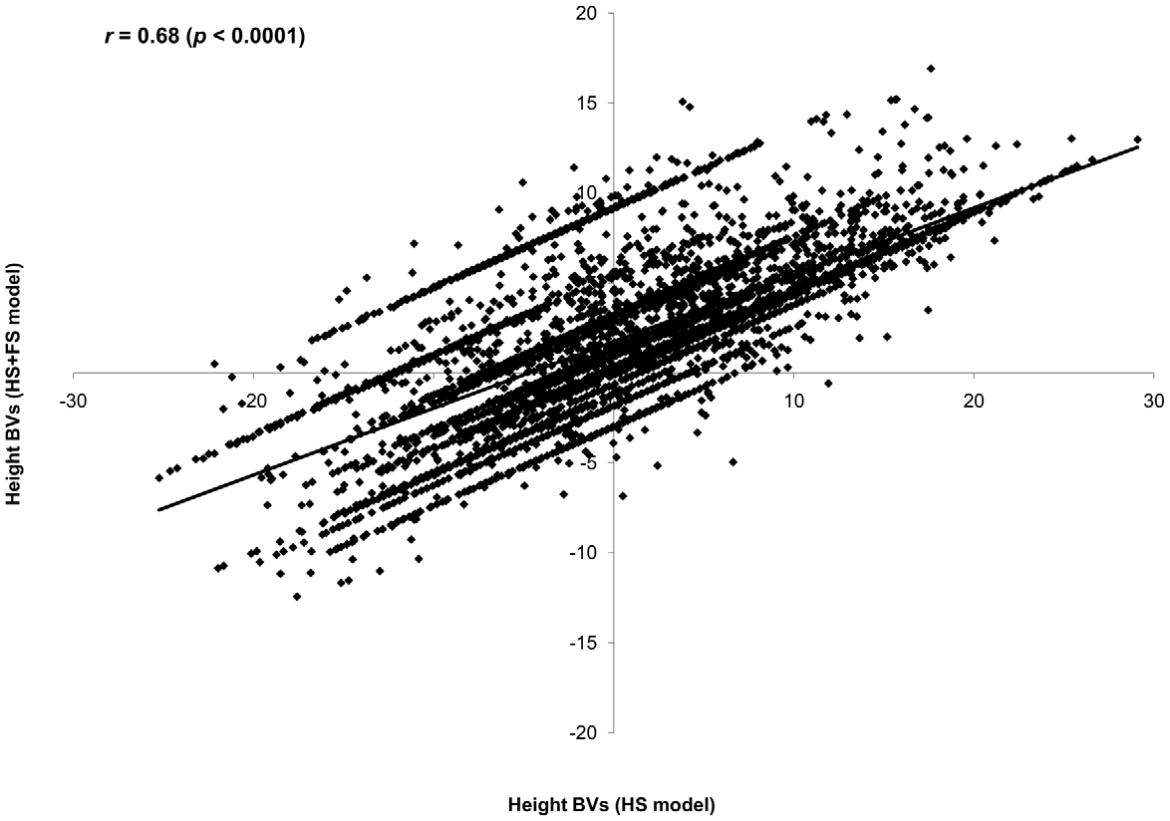
Scatter plot of predicted breeding values for offspring from the two incomplete pedigree models (HS and combined HS+FS). Pearson correlation (*r*) is in the left corner of the graph (note the greater extent of variation between the two models).

Following the classical individual-tree additive model, three analyses were conducted. The first is for the 15 open-pollinated families (HS) with sample size of *N* = 5,796 individuals (i.e., incomplete pedigree). The second is also for the same 15 HS families (*N* = 5,796) but after the inclusion of the male parent for 1,419 individuals (i.e., a combination of half- and full-sib families (HS+FS) and also represents an incomplete pedigree). While the third representing full pedigree (*N* = 1,419) and was solely based on full-sib families formed by the pedigree reconstruction (FS) (Figure 1; Table 1). Relative to the combined HS+FS model, the HS model grossly overestimated the additive genetic variance (156.8 vs. 69.3), which more than doubled the height heritability estimate (0.33 vs. 0.14) (Table 1). The precision of the additive genetic variance (80.0 vs. 26.9) and heritability (0.16 vs. 0.05) estimates for these two models produced higher standard error for the HS as compared to the combined HS+FS model (Table 1). Additionally, the inclusion of more genetic information in the combined HS+FS model (i.e., those from FS families) increased the sensitivity of the analysis, as subtle plot-to-plot variation was detected, resulting in a more realistic assessment of the residual error term (Table 1). Parental breeding values' comparisons was limited to only the 15 maternal parents in the HS analysis with their corresponding 15 estimates from the HS+FS analysis and produced non-significant product-moment (*r* = 0.44 (CI: −0.099, 0.775); *p* = 0.105, Figure 2) and rank (ρ = 0.44 (CI: −0.099, 0.775); *p* = 0.105) correlations. The corresponding comparison of HS with HS+FS breeding values for the offspring yielded significant product-moment (*r* = 0.69 (CI: 0.672, 0.700); *p* = 0.0001, Figure 3) and rank (ρ = 0.67 (CI: 0.656, 0.686); *p* = 0.0001) correlations. Both results clearly demonstrate the reduced utility of the HS model's estimates for forward selection, relative to the results from the HS+FS treatment as indicated by both product-moment and rank correlations. Finally, the average accuracy of predicted breeding values, calculated from the combined HS+FS model was higher for parents (0.81) and offspring (0.55), than their corresponding values (0.56 and 0.45, respectively), calculated from HS model.

**Table 1:** Forth-year height variance components and narrow sense heritability values (h^2^_ns_) and their standard errors for the half-sib (HS), combined half-sib+full-sib (HS+FS) and full-sib (FS) models.

Source of variation	Variance component		
	Incomplete pedigree		Complete pedigree
	HS	HS+FS	FS
Additive	156.8±80.0	69.3±26.9	55.93±25.42
Plot	48.7±11.2	80.7±17.2	101.95±23.93
Error	266.4±60.5	332.4±20.1	315.99±19.52
Total	471.9	482.5	473.9
h^2^_ns_	0.33±0.16	0.14±0.05	0.12±0.05

**Figure 4:**
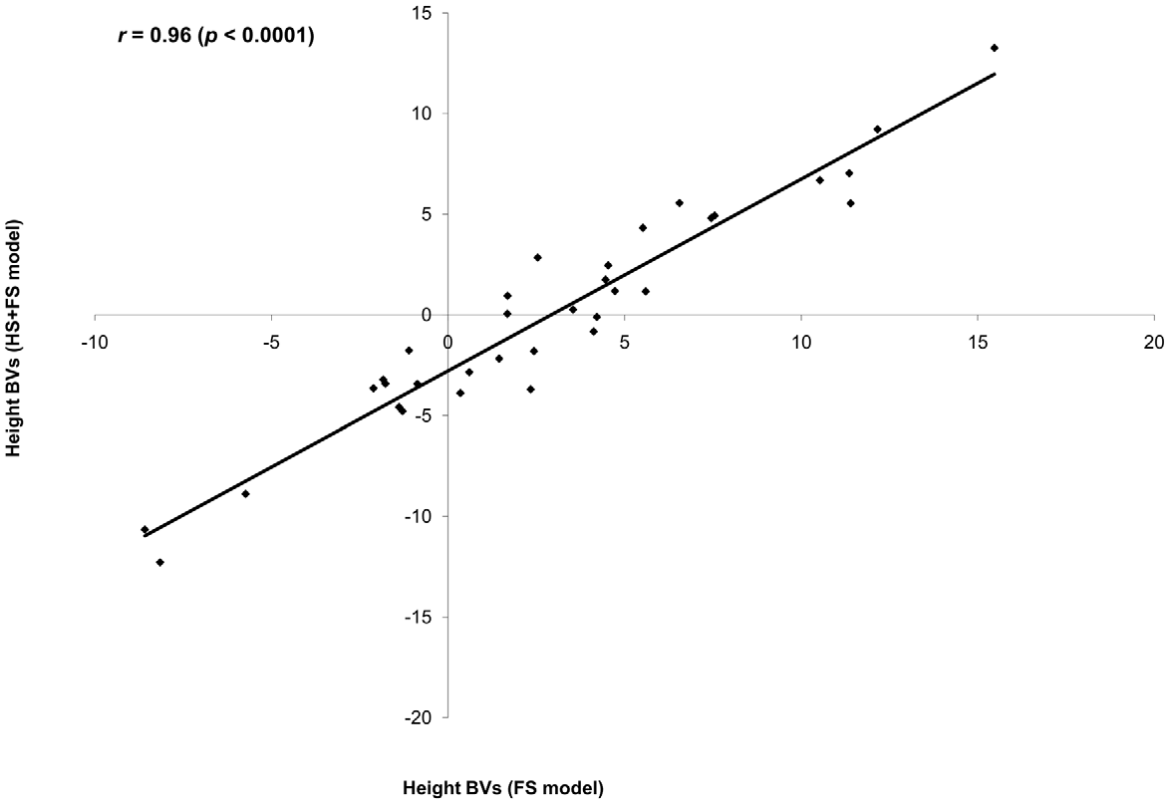
Scatter plot of predicted breeding values for parents from the incomplete (combined HS+FS) and complete (FS) pedigree models. Pearson correlation (*r*) is in the left corner of the graph.

**Figure 5:**
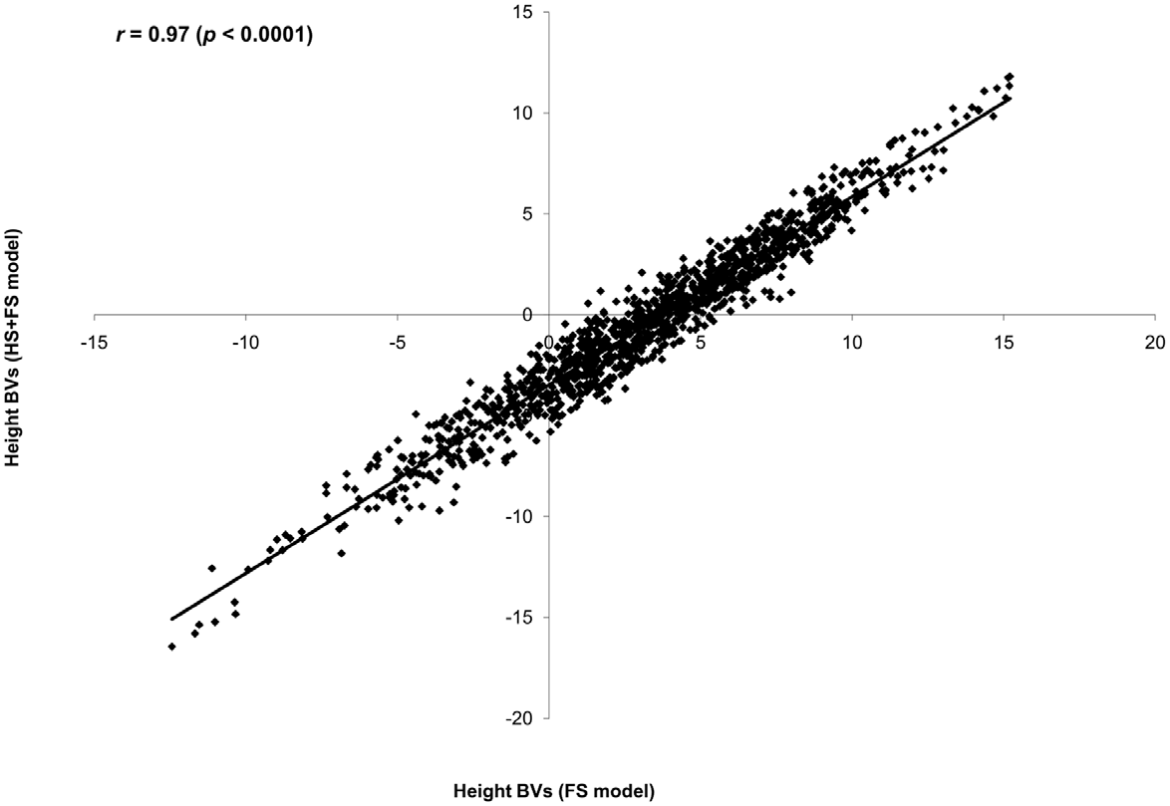
Scatter plot of predicted breeding values for offspring from the incomplete (combined HS+FS) and complete (FS) pedigree models. Pearson correlation (*r*) is in the left corner of the graph.

The full (FS) and combined HS+FS pedigree models produced comparable additive and heritability estimates, with similar precision (Table 1). Predictions of parental breeding values extracted from both models were comparable and highly correlated (product-moment (*r* = 0.96 (CI: 0.928, 0.982); *p* = 0.0001, Figure 4) and rank (ρ = 0.94 (CI: 0.875, 0.968); *p* = 0.0001) correlations). The same was true for offspring breeding values (product-moment (*r* = 0.97 (CI: 0.971, 0.976); *p* = 0.0001, Figure 5) and rank (ρ = 0.97 (CI: 0.967, 0.973); *p* = 0.0001) correlations). The results from the combined HS+FS pedigree approach are robust and reliable. Moreover, the average accuracy of breeding values from parents and offspring calculated from the FS model (0.78 and 0.69, respectively) were very similar to those estimated from the combined HS+FS model (0.76 and 0.64, respectively). It is interesting to note that predicted parental breeding values were produced for the entire parental population (i.e., all seed and pollen donors), even when only 15 maternal parents were used and these estimates were based on the entire population (*N* = 5,796) for the combined HS+FS model as opposed to *N* = 1, 419 for the FS model.

### Production Population Selection

**Figure 6:**
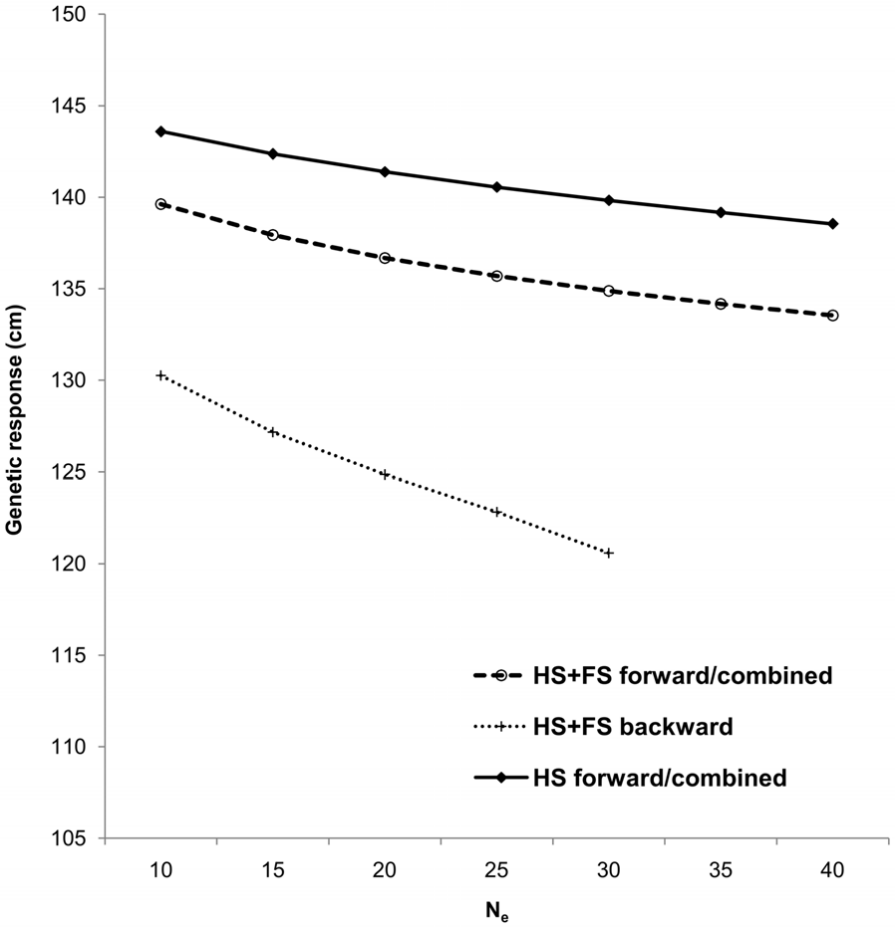
Response to selection comparison between the half-sib (HS) (forward and combined) and combined half- and full-sib (HS+FS) (backward, forward and combined) models assessed across various effective population sizes (10 to 40). (The small number of tested parents resulted in identical results for forward and combined selection methods under the combined HS+FS and HS scenarios).

We implemented three selection options; namely, forwards, backwards, and combined (combination of backwards and forwards), utilizing either the parental (backwards) and/or offspring (forwards) “Best Linear Unbiased Predictors” (BLUPs) generated from the HS or the combined HS+FS models. The backwards selection option was applied exclusively to the combined HS+FS model as parental breeding values were determined from both maternal and paternal information. The limited number of maternal parents (15 seed donors) precluded the application of the backwards selection option under the HS model; however, maternal breeding values along with offspring was used in the HS combined selection. Additionally, the limited number of maternal parents minimized the response to selection's differences between the forwards and combined selections resulting in somewhat identical results (Figure 6). Without exception and across the range of effective population size tested, the HS model overestimated the response to selection as compared to that from the combined HS+FS model, reflecting the observed additive genetic variance overestimation (Figure 6). For example, compared to the combined HS+FS model, the HS combined selection overestimated the response to selection by a range of 15 and 25% for effective population size of 10 and 40, respectively (Figure 6). The combined HS+FS model's forward and/or combined selections were superior to their backward with response to selection differences ranging between 7 and 12% for effective population size of 10 and 30, respectively (the paternal HS family size restriction of n = 6 limited the effective population size range for backward) (Figure 6). Finally, as expected and for all selection methods and both HS and the combined HS+FS models, the response to selection decreased with increased in effective population size (Figure 6).

### Estimating Offspring Optimum Sample Size

**Figure 7:**
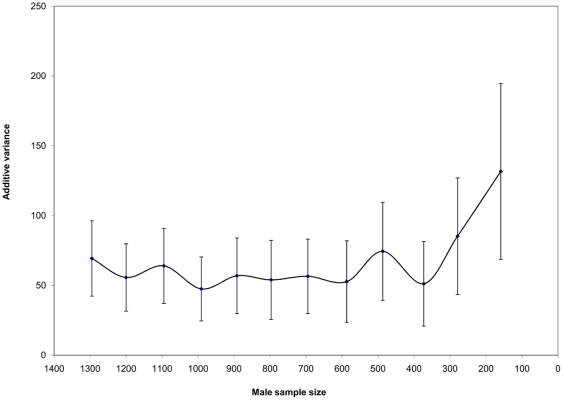
Additive genetic variance estimates as affected by variation in the number of offspring with known male parents. Vertical lines represent the standard error bars for additive genetic variance estimates.

Drastic difference in the additive genetic variance magnitude and its standard error was observed with increasing the number of trees with known paternal information (Figure 7). Increasing the number of trees with known fathers (i.e., those from the pedigree reconstruction) to those already with known mothers improved the direct and/or indirect connectedness among parents and thus permitted their unbiased comparison as well as their genetic parameters' estimation. The observed improvement in the additive genetic variance precision leveled after the inclusion of 600 individuals and no substantial fluctuations were observed beyond this point, indicating that a threshold was reached and the inclusion of any additional offspring would not substantially affect the results (Figure 7). Based on the observed trend and in this particular case, it appears that the inclusion of paternal information for 10% of the evaluated offspring population is adequate to create the direct and/or indirect connectedness among parents is sufficient to achieve the available precision.

## Discussion

The concept of marker-assisted estimation of quantitative genetic parameters was introduced by Ritland [20], whereby traits' heritabilities and the magnitude and direction of their genetic correlations are derived from regressing pair-wise phenotypic similarity on their corresponding pair-wise genetic relatedness. This concept is appealing, because of its obvious simplicity, *in situ* nature (i.e., no experiments or mating designs), and most of all its suitability to long-lived organisms such as trees or wildlife that require long-term experiments or extensive field observations. The distribution of relatedness among the studied individuals is assumption-free, thus it is applicable to natural populations where a vast array of genetic relationships can occur [21], [22]. In situations where offspring are derived from random mating among a set of known parents and more specifically when their number is somewhat limited, the no *a priori* assumption about the expected distribution of genetic relationship becomes inappropriate for a network of full-sibs, half-sibs, and selfs (albeit absence of spatial autocorrelation in relationship coefficients as well as in trait performance in the wild are assumed). It should be stated; however, that the regression approach does not permit the prediction of parents and/or offspring breeding values, thus its application to selection and breeding is somewhat limited.

Conventional tree breeding programs are structured around three main activities: breeding, testing and selection [23]. These activities are long-term endeavours, based on structured pedigree produced from one or a combination of different mating designs [23]; they also require extensive testing in large experimental settings, distributed throughout vast territories [4], and (most important of all), they require sustained organizational and financial commitment. Obviously, simplified breeding schemes that reduce time and cost would be of great value. The generation of complete pedigreed offspring for testing and selection is an obvious target for simplification, fostering incomplete pedigree methods such as open-pollinated family testing [6] and polycross designs [7]. Incomplete pedigree methods, however, are not without their limitations. In particular, open-pollinated testing of the offspring of each maternal parent (seed-donor) assumes they are half-sibs (i.e., sired by different fathers) and non-inbred, and the covariance among half-sib families is assumed to be equal to one-fourth of the additive genetic variance [3]. Both theoretical and empirical studies indicate that this assumption is often violated; as a practical consequence, additive genetic variance is typically overestimated [8]–[10]. The extent of the half-sib assumption violation is expected to be greater if the number of male parents is restricted, as it typically is in a confined breeding population, the usual strategy in breeding arboreta.

To avoid the inaccuracies associated with quantitative genetic parameters assessment from incomplete pedigrees, Lambeth *et al.* [11] proposed the use of molecular genetic markers for paternity assignment, thus converting the incomplete to a complete pedigree, allowing proper genetic parameters estimation and reliable parental and offspring ranking. The same approach was also introduced to open-pollinated testing by Grattapaglia *et al.* [12], who reconstructed the complete pedigrees.

El-Kassaby *et al.* [13] and El-Kassaby and Lstibůrek [17] introduced the concept of “Breeding without Breeding” as a simple, alternative scheme to conventional tree breeding. The method uses: 1) large open-pollinated (i.e., incomplete pedigree) as a primary mean to simplify testing, 2) informative DNA fingerprinting and pedigree reconstruction for a randomly selected subset of the tested individuals to determine their genetic relationship (i.e., complete pedigree) and hence provide adequate bridges between all parents (female and male), 3) the animal model [18] to concurrently analyse the combination of complete (FS: subset) and incomplete (HS: open-pollinated families) pedigree to generate the quantitative genetic parameters needed for selection, and 4) application of an optimization protocol [17] that maximizes the genetic gain at any desired genetic diversity level in a selection scheme. The method capitalizes on the animal model's [18] capabilities of analysing unbalanced and incomplete pedigree to generate the genetic parameters using the “Best Liner Unbiased Prediction” procedure (BLUP [24] needed for parental and offspring evaluation thus facilitating backwards, forwards, or a combined (backwards and forwards) selection in a breeding framework. Therefore, the fundamental difference between the assembled genetic relationship among individuals in the BwB scheme (present study) and those from either the polycross [11], [16] or open-pollinated testing [12]–[16] is that the former does not require complete pedigree for the tested population (a combination of large half-sibs and several smaller full-sibs families) while the latter explicitly stipulates the availability of complete pedigree information for every individual for quantitative genetic parameters estimation.

Quantitative genetic parameters comparison between the two incomplete-pedigree models (i.e., HS and the combined HS+FS) indicated that the HS model over-estimated the additive genetic variance and its surrogate heritability and under-estimated the environmental effects (Table 1). As expected, the genetic relationships (half-sib, and full-sib; Figure 1) within the studied 15 half-sib families should have reduced the average covariance among relatives within the HS model, thus the resulting additive genetic variance is unrealistically inflated. Furthermore, the HS model failed to detect the subtle site heterogeneity present in the experimental site [25], hence the observed under-estimation of the plot effect (Table 1). This is due to the fact that the 15 half-sib families were present in 4 large, 10×10 replications which made it difficult to definitively separate the genetic and environmental effects within experimental units (i.e., plots). In multiple-tree and contiguous plots designs, substantial environmental covariance among family members is confounded with genetic covariance of a given plot [25]. The degree of confounding depends on the size of the plots and the patterns of environment variability. In general, the larger the plot, the more difficult it is to cleanly separate genetic from environmental effects. On the other hand, site heterogeneity was clearly detected after the inclusion of more genetic information in the combined HS+FS model (i.e., those resulting from the pedigree reconstruction of 1,419 individuals which resulted in a better site heterogeneity detection due to their presence across all half-sib families and their respective replications). It is noteworthy to mention that the changes in variance components apportionment over the HS and the combined HS+FS models' sources of variation, collectively affected the resulting heritability estimate (Table 1). While it is only for a subset of the offspring, the inclusion of additional paternal information in the combined HS+FS model permitted covariance among relatives adjustment and hence the observed improvement in the generated parameters, a situation cannot be attained under the HS model (i.e., open-pollinated test). The discrepancy between the two models is further demonstrated by the low to moderate correlations between either paternal or offspring breeding values (Figs. 2–3) and their different average accuracy of prediction (0.56 vs. 0.81 for parents and 0.45 vs. 0.55 for offspring), highlighting the reduced reliability of the open-pollinated testing for either backwards or forwards selection. Furthermore, the combined HS+FS model allowed predicting the breeding value for the entire parental population (38 vs. 15) as it utilized all offspring information irrespective of parental gender (i.e., as pollen and/or seed donors) while the HS model was restricted only to the maternal population (i.e., seed donors).

The observed differences between the two incomplete pedigree models (HS and the combined HS+FS) support the beneficial role of including the pedigree reconstruction information even though it is only from a subset of the studied population. The inclusion of additional genetic information allowed the creation of linkages among the 15 half-sib families (known seed donors) with all parents participated in mating (pollen donors), thus increasing the sample size (i.e., higher genetic parameters' precision and breeding values' accuracy) and maximizing the BLUP-method utilization for breeding values prediction (see Ronningen and Van Vleck [24], for detailed explanation). The comparison between the combined HS+FS and full pedigree (FS) models is also needed to illustrate the advantages of partial pedigree inclusion. The full pedigree (FS) model is based on the assembled mating design from the pedigree reconstruction that is based on 1,419 offspring. Variance components and their precision and parental and offspring breeding values comparison between the two models produced similar estimates (Table 1; Figs. 4–5) and accuracies for parents (0.76 vs. 0.78) and offspring (0.64 vs. 0.69) were virtually identical. Heritability estimates are known to be population-specific [3]; however, the two models produced comparable 4-year height heritability estimates (HS+FS: 0.14±0.05; FS: 0.12±0.05), indicating similar magnitude/trajectory. This is not surprising since the two populations share 1,419 individuals in common and the combined HS+FS model included additional 4,258 individuals with known maternal parents. More importantly, the striking similarity between parental and offspring breeding values between the two models are indicative of similar ranking even though different number of individuals and genetic information were used. The observed high correspondence between the suggested combined HS+FS and complete pedigree models highlights the superiority of the proposed BwB [17] indicating that a mixture of incomplete (half-sibs) and complete (full-sibs) pedigree is an efficient approach for acquiring reliable quantitative genetic parameters. The fingerprinting of a subset of the testing population is expected to substantially reduce the cost associated with pedigree reconstruction without any parameters' precision penalties.

The advantage of the combined HS+FS model over the HS and/or FS models is clearly demonstrated at the selection stage (Figure 6). Notwithstanding the overestimation of the additive genetic variance, the HS model is restricted to backward selection from the studied female parents as no BLUP values are generated for their male counterparts (i.e., 15 out of 38). The FS model is better than the HS as it allows the generation of accurate BLUP values for the 38 parents participated in mating as well as their offspring (N = 1,419) which is a subset of the tested population (N = 5,796), thus limiting forward selection to the fingerprinted offspring and thus does not consider any of the non-fingerprinted offspring which represent a substantial part of the tested population (57%). The combined HS+FS model, on the other hand, provides BLUP values for the parents and their offspring, irrespective of their family status, thus increasing the efficiency of forwards selection and improving the precision of backwards selection as well as combined selection. Additionally, the establishment of open-pollinated vs. those based of full pedigree field tests is more simplistic and can be effectively done with reduced efforts and cost.

The large number of parents commonly tested in traditional tree improvement programs requires the use of “efficient” mating designs so manageable number of crosses are made (e.g., disconnected partial diallel [4], [23]). In these mating designs, the parental population is divided to multiple subsets of parents with crosses are often restricted to within parental subsets with minimal or no matings among members of the different subset, thus creating opportunities for genetic sampling (i.e., no opportunity for cross referencing across set). The present study has demonstrated that paternity assignment of wind-pollinated half-sib families from know seed-donors provided adequate linkage across parents, hence we propose the implementation of similar approach concurrently with the selected traditional mating schemes to provide means for cross referencing and the avoidance of genetic sampling.

If BwB is to be considered as a viable option for tree breeding, then several additional questions must to be answered, among them: 1) what is the proportion of the population needed for pedigree reconstruction? 2) What is the minimal HS and/or FS family size required for proper BLUP analysis? 3) What is the role of elevated gene flow or selfing in the breeding population? 4) How many parents can be realistically tested? 5) How are we to expand testing beyond those parents present in the breeding population? The observed changes in the additive genetic variance estimates and their associated precision that accompany changes in the number of genotyped individual with known male parents (i.e., those resulting from the pedigree reconstruction) suggest that the inclusion of approximately 10% of the tested population is adequate to reach stable parameter estimates (Figure 7). The main function of these individuals is to create enough connections between parents, thus permitting direct and/or indirect comparison among the parental population members, a fundamental prerequisite for the BLUP analysis [24]. Increasing the number of offspring with known fathers to those already with known mothers increased the direct and/or indirect connectedness among parents and thus permitted their unbiased comparison as well as the estimated genetic parameters. Rönningen and Van Vleck [24] explicitly stated that a minimum of two offspring between any two males is needed for proper parameters estimation. In the present analysis, we imposed a minimum half-sib family size of six for any parent to be included and the observed correspondence between parents and offspring breeding values between the combined HS+FS and FS models is a reflection of this practice. The number of offspring designated for fingerprinting will also be affected by the degree of gene flow. As gene flow increases, more genotyped individuals will not provide any paternal information for connecting the different parents, but those individuals will remain in the analysis if they are among the maternal parents evaluated. Additionally, as the selection differential between the gene flow's source and the parental breeding population increases, the greater the difference in their offspring performance. A simple offspring phenotypic ranking followed by truncation selection theoretically could eliminate a substantial amount of the inferior offspring [17]. Offspring produced through selfing, while limited, remained in the data analysis through the inclusion of the pedigree information, and thus the estimated genetic parameters should be minimally affected. The rate of selfing among the tested parents is expected to provide an idea of the selfing propensity variation, which may shed some light on the relationship between selfing rate and general combining ability. As the number of parents' increases, the number of informative genetic markers must increase to allow for the exclusion power needed for pedigree reconstruction. The use of paternally inherited markers such as cpDNA could aid in differentiating among males with similar autosomal multilocus genotypes. Increasing the number of marker loci and including paternally inherited markers is expected to increase the experimental efforts; however, the increased efforts should be evaluated in light of the number of parents tested. Finally, increasing the number of tested parents beyond what is present in the breeding population could be accomplished through the use of supplemental-mass-pollination, a technique known to successfully incorporate pollen from specific parents in natural wind pollination of unprotected receptive females [26].

## Materials and Methods

### Plant material

In 2005, wind-pollinated seed samples from 15 unrelated parents were collected from a 41-parent western larch (*Larix occidentalis* Nutt.) seed orchard. The sampled orchard is one of two genetically distinct (41 and 62 parents) orchards established by British Columbia Ministry of Forests, Lands and Natural Resource Operations to provide genetically improved seed to the Nelson (<1,300 m) and East Kootenay (800–1,500 m) seed production units. These orchards are located near Vernon, B.C., Canada (altitude 480 m, latitude 50°14′N, longitude 119°16′E), in an area devoid of western larch background pollen. The orchards are separated by an 8 m wide road and a row of black cottonwood (*Populus* trichocarpa Torr. & Gray) trees, acting as a partial pollen barrier. Seed samples and orchard's reproductive survey data were provided by British Columbia Ministry of Forests, Lands and Natural Resource Operations as the orchard is part of a co-operative arrangements among government-private industry-academia. Seed were sown (February, 2006) by individual maternal family in a commercial nursery in growing blocks (80 cavities/block), soil mixes, irrigation, heating, and fertilization regimes similar to those operationally applied for reforestation seedling production. Seed pre-treatment (i.e., pre-chilling to break dormancy) prior to sowing followed International Seed testing Association procedures [27]. At the end of the growing season (September, 2006), seedlings were extracted, by family, and used to establish a common garden trial.

### Common garden trial

The trial was established at the University of British Columbia's Research Facility (latitude 49° 15′N, longitude 123° 15′W, elevation 79 m), laid out as a randomized complete design with four replications. Each replication consisted of 10×10 square plots at a spacing of 0.3×0.3 m (100 seedlings/family). At the end of the third field growing season (fall of 2009, 4 years from germination), total seedling heights (HT in cm) were measured on all surviving trees (5,306). The trial was watered and weeded when needed, and survival was 88% at the time of height measurement.

### DNA fingerprinting and paternity assignment

The two orchards (studied and neighbouring, with their 41 and 62 parents, respectively) represent the possible paternal parents for a randomly selected 1,538 offspring that were genotyped with 16 microsatellite (SSR) markers. The SSR markers used were: 1) seven developed for *Larix occidentalis* [28], 2) two developed for *L. lyalli* [29], with one primer (UAKLly13) amplifying two loci (UAKLly13-1 and UAKLly13-2) in *L. occidentalis*, and [3] seven developed for *L. kaempferi* [30] (Table S1). Touchdown PCR was performed according to the protocol used by Isoda and Watanabe [30]: 94°C for 1 min followed by 10 cycles/30 s at 94°C, 30 s/63°−53°C (−1°C at each cycle) for 1 min, followed by 25 cycles of 30 s/94°C, 30 s/53°C and 1 min/72°C followed by 10 min/72°C. The CERVUS program ver. 3.0.3 was used to estimate null allele frequencies in the studied orchard's parental population [31]–[32], as null alleles introduce errors in parentage analysis by leading to high frequencies of false parentage exclusions [33]. PCR multiplexing was developed for four sets of loci sharing the same annealing temperature: 1) UBCLXdi-16, UBCLX1-10, and UBCLXtet-21, 2) UBCLXtet_2-12, UAKLly10, and UAKLly13, 3) bcLK33, bcLK66, bcLK211, and bcLK258, and 4) bcLK232 and bcLK263 (Table S1). Our preliminary paternity analysis, showed a 10% increase of paternity assignment after removing SSR loci with high null allele frequencies, but we included UBCLXtet-21 in spite of its high null allele frequency, because it was easy to multiplex and score as tetra-nucleotide SSR. Additionally, our results showed that the inclusion of this locus did not introduce serious parentage assignment bias. In total, 10 SSR loci were used for parentage assignment (Table S1). After paternity assignment (below), 98% of genotyped offspring were sired by members of the two orchards' panel of fathers. The CERVUS program [31], [32] provides likelihood based paternity inference with a known level of statistical confidence that accounts for genotyping error; we used it to to assign the pollen donor for 1,538 offspring. A genotyping error rate of 0.03 across the 10 loci was estimated from the known mother-offspring genotypes (Table S1). The paternity assignment was based on 10,000 simulations, with the 41-parents as candidate fathers. The log-likelihood (LOD) score, the likelihood that the candidate parent is the true parent divided by the likelihood that the candidate parent is not the true parent, was calculated for each putative parent. The delta score, the difference in LOD scores of the two most likely candidate parents, was used as a criterion for assignment of parentage at the 95% level of confidence in our analysis.

### Quantitative genetics analyses

A classical individual-tree additive model, assuming no dominance and epistatic effects, was used. The model included a fixed effect of overall mean (**β**), a normally distributed random additive genetic effect (***a***, breeding values), with covariance matrix ***A*** = {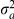} where ***A*** is the additive relationship matrix (see below [34]) among all trees: parents without records, plus offspring with data, and 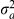 the additive genetic variance. The model also included a normally distributed random plot effect term (***p***) with mean zero and variance 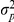. Finally, a normally distributed random error (***e***) with mean zero and variance 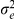 were included. Let ***y*** be a vector containing the tree individual observations for height. Then, in matrix notation, the classical individual-tree additive model can be described as:
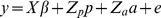
(1)


Let ***A*** be the additive relationship matrix based on pedigree. The ***A*** matrix has diagonal elements equal to 1+*F_i_*, where *F_i_* is the inbreeding coefficient for the *i* ^th^ individual and off diagonals equal to the additive relationships ***A*** *_ij_* between tree *i* and *j*. Three individual-tree additive mixed models (model 91)) were evaluated using different pedigree files. Assuming that parent trees were unrelated, the first model, half-sib (HS model), was used with the known female parent of each individual, where all individuals are assumed not inbred (i.e., *F_i_* = 0), and the additive genetic relationship are 0.25 or 0.0 for both trees with different fathers (with unrelated pollen), thus being maternal half-sibs and unrelated trees, respectively. This model is commonly used by forest geneticists and is called the open-pollinated test, where individuals within an open-pollinated family are assumed to be half-sibs [8]. The pedigree reconstruction created two more scenarios, one includes known female parents for all individuals in the common garden (the sampled 15 seed donors) and the male parents (any one of the orchard's 41 parents) for those used in the pedigree reconstruction (1,419 seedlings) (combined HS+FS model). The second includes only the 1,419 seedlings with their known maternal and paternal parents (known as the FS model). When male parents are known, correct inbreeding coefficient (i.e., *F_i_* = 0.5) and additive relationship between trees ranging from selfs to half-sibs (e.g., ***A*** *_ij_* = 1 if two individuals are generated by self-pollination or ***A*** *_ij_* = 0.5 if two individuals are full-sibs through a common father) were considered in the ***A*** matrix.

### Variance components

Restricted Maximum Likelihood (REML [34]) was used to estimate variances for the random effects of the classical individual-tree additive model (model (1)) and was obtained with the ASReml program [35], which uses the average information algorithm described by Gilmour *et al.* [36]. The narrow-sense individual heritability (*h* ^2^) was calculated as 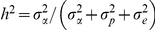, where 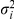 with *i* = *a*, *p*, and *e* are the values of the additive, plot, and error variance of the individual-tree model (1). Additionally, the inclusion of male information in the pedigree matrix allowed expanding model (1) to estimate the additive genetic variance after considering the additional genetic relationships generated by pedigree reconstruction. This was done to allow comparing the classical individual-tree additive models used. An important limitation of the REML (co)variance estimates is that their distribution is unknown. Only an approximate measure of precision of the estimates based on asymptotic or large sample theory can be calculated. Approximate standard errors (s.e.) of the 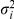 and *h* ^2^ were computed with the “delta method” based on the Taylor expansion [18] using ASREML [35].

### Prediction of the breeding values and response to selection

The analysis of a progeny test normally involves two steps: first the estimation of variance components and second the prediction of breeding values for the individuals, using the variance components estimated in the first step. In the three models, the “Best Linear Unbiased Predictors” (BLUPs) of parent and offspring breeding values were computed with ASReml from the estimated variance components. The accuracy of the predicted breeding values was calculated using the following expression: 
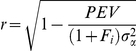
. The acronym PEV stands for ‘prediction error variance’ [36] of predicted breeding values, using the BLUPs of parent and offspring and *F_i_* is the inbreeding coefficient for the *i* ^th^ individual. The PEV is calculated as the diagonal elements of the inverse of the coefficient matrix from the mixed model equations [36]. To make the accuracies comparable across models (i.e., HS, combined HS+FS and FS), the variance components required to set up the mixed model equations were those estimated from the combined HS+FS. Pearson product-moment correlation and Spearman rank-order correlation were also calculated to compare whether the strength of linear dependence and the ranking of predicated breeding values differed among models. Additionally we have included confidence intervals of all correlation estimates to evaluate jointly the variance and sample size under the alpha value of 0.05. Individual tree BLUP values were used to compare the response to selection under the HS (forward and combined) and combined HS+FS (backward, forward and combined) models, as affected by effective population size, using the optimization protocol outlined in El-Kassaby and Lstibůrek [17].

### Estimating offspring optimum sample size

To determinate the optimum number of individuals with known fathers needed for obtaining reliable genetic parameters and thus reducing the DNA fingerprinting efforts, the classical individual-tree additive model (1) was fitted with several pedigree files, where the male information was randomly and progressively deleted, thus increasing percentage of omitted male data from 7 to 92% (i.e., reducing the number of individuals with known male parents). These pedigrees with randomly deleted males provided us with a range of values and standard errors associated with them that the different parameters may take and permitted us to investigate the robustness of results under reduced fingerprinting efforts (i.e., reduce the number of offspring with known paternal parents). For this data set, we set the minimum paternal HS family to n = 6 for inclusion in the analyses and hence the generation of precise genetic parameters and their respective predicted breeding values.

## Supporting Information

Table S1 Annealing temperature in °C, number of alleles, observed (*H*_o_) and expected (*H*_e_) heterozygosities, and estimated frequencies of null alleles and genotyping error of the seed orchard population used in the present study (41-Parents).
(DOCX)
