# Supplementary material for: Breeding without Breeding: Is a Complete Pedigree Necessary for Efficient Breeding?
Source: PLoS One. 2011 Oct 3;6(10):e25737. doi: 10.1371/journal.pone.0025737 (PMC3185014; doi:10.1371/journal.pone.0025737)
Supplement: Table S1 — Annealing temperature in °C, number of alleles, observed (H o) and expected (H e) heterozygosities, and estimated frequencies of null alleles and genotyping error of the seed orchard population used in the present study (41-Parents). (DOCX) [file pone.0025737.s001.docx]

**Table S1.** Annealing temperature in °C, number of alleles, observed (*H*_o_) and expected (*H*_e_) heterozygosities, and estimated frequencies of null alleles and genotyping error of the seed orchard population (41-Parents).

| Locus | Annealing  temperature | No. | Heterozygosity | | Null allele  Frequency | Genotyping  error rate |
| --- | --- | --- | --- | --- | --- | --- |
|  |  | alleles | *H*_o_ | *H*_e_ |  |  |
| UBCLXtet_2-11^a^ | 58 | 8 | 0.488 | 0.528 | 0.0536 | 0.0200 |
| UBCLX1-10^a^ | 58 | 8 | 0.700 | 0.733 | 0.0183 | 0.0601 |
| UBCLXtet-21^a^ | 58 | 8 | 0.550 | 0.810 | 0.1802 | 0.1225 |
| UAKLly10a^b^ | 58 | 14 | 0.902 | 0.877 | 0.0223 | 0.0287 |
| UAKLly13-1^b^ | 58 | 11 | 0.683 | 0.773 | 0.0557 | 0.0072 |
| UAKLly13-2^b^ | 58 | 7 | 0.585 | 0.560 | 0.0336 | 0.0092 |
| bcLK066^c^ | 63 → 53 | 6 | 0.846 | 0.783 | 0.0459 | 0.0359 |
| bcLK211^c^ | 63 → 53 | 4 | 0.605 | 0.598 | 0.0181 | 0.0277 |
| bcLK253^c^ | 63 → 53 | 9 | 0.700 | 0.729 | 0.0211 | 0.0033 |
| bcLK258^c^ | 63 → 53 | 10 | 0.703 | 0.769 | 0.0456 | 0.0058 |
|  |  |  |  |  |  |  |
| UBCLXA4-1^a^ | 62 | 7 | 0.634 | 0.740 | 0.0742 | NA^d^ |
| UBCLXdi-16^a^ | 58 | 9 | 0.267 | 0.812 | 0.5069 | NA |
| UBCLXdi-21^a^ | 55 | 8 | 0.400 | 0.698 | 0.2511 | NA |
| UBCLXtet_2-12^a^ | 58 | 4 | 0.152 | 0.634 | 0.6103 | NA |
| bcLK033^c^ | 63 → 53 | 6 | 0.389 | 0.649 | 0.2434 | NA |
| bcLK232^c^ | 63 → 53 | 8 | 0.463 | 0.614 | 0.1653 | NA |
| bcLK263^c^ | 63 → 53 | 9 | 0.590 | 0.832 | 0.1549 | NA |

^a^Chen *et al.* [28]; ^b^Khasa *et al.* [29]; ^c^Isoda and Watanabe [30].

^d^NA, data not available for these loci due to their exclusion from paternity assignment.
